# Development of AlN and TiB_2_ Composites with Nb_2_O_5_, Y_2_O_3_ and ZrO_2_ as Sintering Aids

**DOI:** 10.3390/ma10030324

**Published:** 2017-03-22

**Authors:** José C. González, Miguel Á. Rodríguez, Ignacio A. Figueroa, María-Elena Villafuerte-Castrejón, Gerardo C. Díaz

**Affiliations:** 1 Facultad de Ciencias Químicas e Ingeniería, Universidad Autónoma de Baja California, Calzada Tecnológico 14418, Mesa de Otay, Tijuana 22390, Mexico; diazg@uabc.edu.mx; 2 Instituto de Cerámica y Vidrio, CSIC, C/Kelsen 5, Campus de Cantoblanco, 28049 Madrid, Spain; mar@icv.csic.es; 3 Instituto de Investigaciones en Materiales, Universidad Nacional Autónoma de México, Ciudad Universitaria, A.P. 70-360, Ciudad de México 04510, Mexico; iafigueroa@unam.mx (I.A.F.); mevc@unam.mx (M.-E.V.-C.)

**Keywords:** park plasma sintering, composites, mechanical properties

## Abstract

The synthesis of AlN and TiB_2_ by spark plasma sintering (SPS) and the effect of Nb_2_O_5_, Y_2_O_3_ and ZrO_2_ additions on the mechanical properties and densification of the produced composites is reported and discussed. After the SPS process, dense AlN and TiB_2_ composites with Nb_2_O_5_, Y_2_O_3_ and ZrO_2_ were successfully prepared. X-ray diffraction analysis showed that in the AlN composites, the addition of Nb_2_O_5_ gives rise to Nb_4_N_3_ during sintering. The compound Y_3_Al_5_O_12_ (YAG) was observed as precipitate in the sample with Y_2_O_3_. X-ray diffraction analysis of the TiB_2_ composites showed TiB_2_ as a single phase in these materials. The maximum Vickers and toughness values were 14.19 ± 1.43 GPa and 27.52 ± 1.75 GPa for the AlN and TiB_2_ composites, respectively.

## 1. Introduction

Aluminum nitride (AlN) ceramics have attracted growing interest in recent years for their high thermal conductivity and good electrical properties. Their thermal conductivity varies between 80 and 260 W/m/K. They exhibit good electrical insulation and have a low dielectric constant (9 at 1 MHz), high thermal conductivity and a low thermal expansion coefficient (4.4 × 10^−6^ K^−1^). AlN-based materials have a wide field of applications in structural and refractory areas [1]. However, because of the high degree of covalent bonding in AlN, good densification of the material is difficult and liquid-phase sintering is fully necessary [2].

AlN ceramics with good densification can be prepared at high sintering temperatures and with long sintering times. This not only increases the production costs but also promotes significant grain growth, which results in the deterioration of the mechanical properties. A typical feature of the densification process is the addition of a limited fraction of sintering additives, rare earth and/or alkaline earth oxides, to promote densification [3,4]. These additives have a dual role during sintering; one could be helping the formation of the liquid phase, which can promote densification through liquid-phase sintering. The other is to improve the thermal conductivity by decreasing the oxide impurities in the AlN lattice [5].

AlN particles always contain some Al_2_O_3_ oxide on the surface (~1%), which reacts with the Y_2_O_3_ sintering additive and AlN powder, forming a liquid phase. The reaction between Al_2_O_3_ and Y_2_O_3_ generates a liquid-phase yttrium aluminate garnet Y_3_Al_5_O_12_ (YAG), yttrium-aluminum perovskite YAlO_3_ (YAP), and yttrium aluminum monoclinic Y_4_Al_2_O_9_ (YAM), which promote the liquid-phase sintering of AlN during the process [6].

The reaction in the AlN-ZrO_2_ system results in a combination of Al_2_O_3_, ZrN, ZrO_2_, and AlN, depending on the heat treatment conditions. A quaternary Zr-Al-O-N phase has also been identified in the AlN-ZrO_2_ system [7]. In the case of the AlN-Nb_2_O_5_ system, NbN, Nb_2_N, Nb_3_N, and Nb_4_N_3_ phases have been identified [8].

Titanium diboride (TiB_2_) is a material of growing interest among the various ultra-high-temperature ceramics (UHTC) due to its characteristic high melting point (~3225 °C), low density (4.5 g/cm^3^), high hardness (25 GPa), good thermal conductivity (96 W/m/K), high electrical resistance (22 × 10^6^ Ω·cm), low thermal expansion coefficient (7.4 × 10^−6^ K^−1^) and high wear resistance [9]. These excellent properties makes it attractive for many high-temperature structural applications such as cutting tools. The densification of monolithic TiB_2_ requires extremely high sintering temperatures of up to ~2100 °C [10] and long holding times due to the predominance of covalent bonding and the low self-diffusion coefficient. Such extreme processing conditions result in exaggerated grain growth of the as-sintered materials, leading to degradation of the mechanical properties [11,12].

TiB_2_ with ZrO_2_ is stable at high temperature. ZrO_2_ is one of the most used oxides in the preparation of TiB_2_-ZrO_2_ systems because it exhibits excellent mechanical properties such as hardness and fracture toughness [13,14,15]. The high toughness of the zirconia monoliths originates from the stress-induced transformation of the stabilized tetragonal phase in the stress field of propagating cracks, a phenomenon known as transformation toughening [16].

One way to sinter these materials is through an advanced sintering method such as spark plasma sintering (SPS). During the SPS process, by virtue of special heat effects such as joule heat, electromagnetic field and electrical discharge, highly densified ceramics are obtained at relatively low temperatures in a very short sintering time and with uniform heating for sintered bodies. Functional materials, ceramics, cermets, intermetallic compounds, and so on [17] have been processed by this method.

In this study, for the first time, composites of AlN and TiB_2_ were sintered by SPS to evaluate the applicability of SPS techniques in compacting the AlN and TiB_2_ composites. The effect of the Nb_2_O_5_, Y_2_O_3_ and ZrO_2_ contents on the mechanical properties and the densification of composites were studied in detail.

## 2. Results and Discussion

Table 1 shows the theoretical and experimental densities of the AlN and TiB_2_ composites obtained by SPS at temperatures of 1800 °C and 1950 °C, respectively. The results for the sintering process showed that the relative density of most prepared composites was above 96%. The lowest density obtained was 92.2% for the TiB_2_-ZrO_2_ (TZ) composition; this could be attributed to the tetragonal/monoclinic transformation during cooling. This process can induce sample fracture due to volume expansion and the refractoriness of zirconia tends to minimize the amount of liquid phase during sintering [13,14,15,16].

### 2.1. AlN Composites

Xiong et al. [3] reported a relative density of 99.8% by SPS of AlN at 1800 °C, 10 min, 30 MPa, while using 3 wt % Y_2_O_3_. It is difficult to obtain high-density AlN ceramics at low temperatures using conventional sintering methods. The relative density of a pure AlN ceramic fabricated by a pressureless sintering method was 75% when sintered at 1800 °C for 3 h, and fully dense AlN ceramics could be obtained at above 1700 °C with long soaking times with sintering additives using hot-press sintering [18]. These comparisons clearly indicate that superior densification can be achieved through the SPS process. The magnitudes of the densities observed are likely to be related to the competition between densification and grain growth. Here, the results show that there is no improvement in the density with increasing the time or sintering pressure.

#### 2.1.1. Crystalline Phases

**Figure 1:**
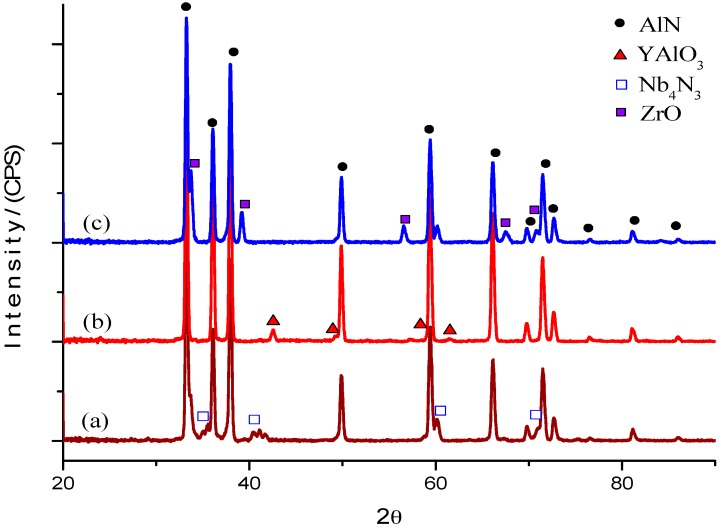
XRD patterns of sintered AlN samples with different oxides: (**a**) AN; (**b**) AY; (**c**) AZ.

Figure 1 shows the X-ray diffraction (XRD) patterns of the AlN-based composites after sintering at 1800 °C. All XRD diffraction patterns were obtained from the polished surface of the specimen. The results of XRD showed that AlN is the main phase for all samples. When Nb_2_O_5_ is used as a sintering aid, it reacts with the AlN to generate Nb_4_N_3_, as reported by Chumarev et al. [8]; at high temperatures, Nb_2_O_5_ is reduced to NbN and Nb_2_N. The Nb_4_N_3_ phase may result from the following reaction:

2NbN + Nb_2_N → Nb_4_N_3_ (1)

Nb_4_N_3_ was found to be a grain boundary secondary phase in AlN-Nb_2_O_5_ (AN). According to the literature, Y_2_O_3_ reacts with alumina (Al_2_O_3_) on the surface of the particles of AlN to generate a precipitate with the phase of yttrium aluminate, then forms a glass phase in the grain boundary as yttrium-aluminum garnet Y_3_Al_5_O_12_ (YAG), yttrium-aluminum perovskite YAlO_3_ (YAP), and yttrium aluminum monoclinic Y_4_Al_2_O_9_ (YAM) [18]. The type of the remaining phases depends on the ratio of the amount of oxygen content in the starting AlN powder and the amount of Y_2_O_3_. The YAP phase was found to be a grain boundary secondary phase in AlN-Y_2_O_3_ (AY). In the AlN-ZrO_2_ (AZ) sample, the grain boundary secondary phase was ZrO according to JCPDS card number 01-089-4768.

#### 2.1.2. Microstructural Analysis

**Figure 2:**
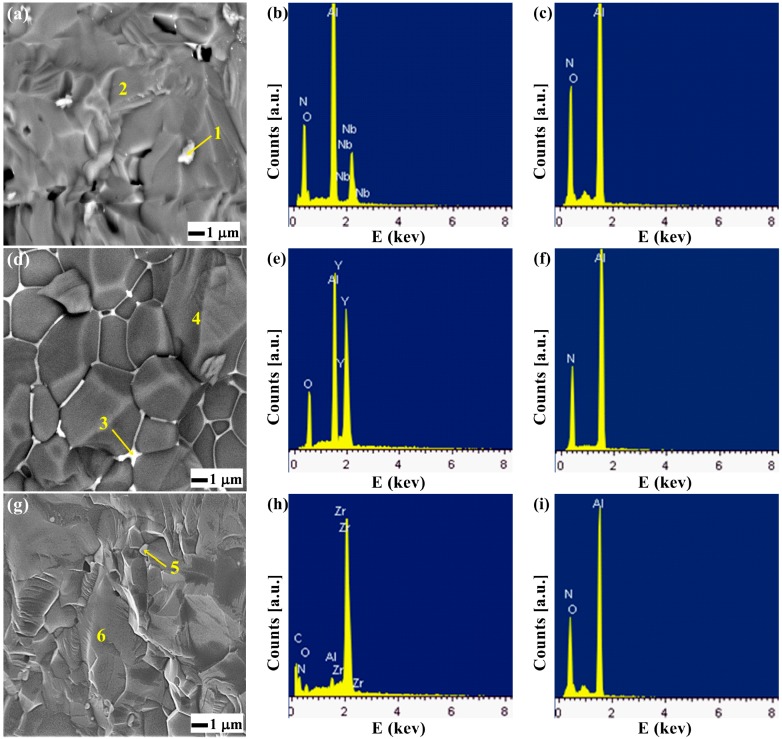
Secondary electrons SEM images of fracture surfaces of AlN specimens sintered: (**a**) AN; (**d**) AY; (**g**) AZ; EDS spectra of the secondary phases: (**b**) analysis of area 1; (**e**) analysis of area 3; (**h**) analysis of area 5; (**c**,**f**,**i**) are from the matrix grains.

Figure 2 shows the scanning electron microscopy (SEM) images of fracture surfaces of AlN specimens at 1800 °C and the microanalyses of energy dispersive X-ray spectrocopy (EDS) of the secondary phase in AN, AY and AZ composites.

The microstructures are mainly composed of AlN grains and the grain boundary secondary phase. To understand the interface between the grain boundary secondary phase and the matrix of AlN, EDS analysis was performed. Figure 2c,f,i show the EDS analyses of the matrix grain of the samples. AN, AY and AZ consist of Al, N and O elements, according to the AlN formula, which is consistent with the XRD result (Figure 1). As shown, the microstructures are composed of AlN grains and the grain boundary secondary phase [19]. Figure 2b,e,h show the EDS analyses of the white part in the grain boundary secondary phase. It is necessary to note that the grain boundary secondary phase of the AY composite remains in the grain boundary, which is probably not good for its thermal behavior. The Nb_4_N_3_ phase was found in AN, the YAP phase was found in AY, and the ZrO phase was found in AZ.

#### 2.1.3. Mechanical Properties

The mechanical properties (Table 2) of the composites were compared with the hardness of monolithic AlN (10.6 GPa). The hardness increased approximately 34% in the AN, 8% in the AY and 33% in the AZ samples. The measured toughness values, obtained with an indentation load of 20 kg, are shown in Table 2. Zhang et al. reported a fracture toughness of 3.2 MPa·m^1/2^ for monolithic AlN ceramics sintered at 1600 °C with a holding pressure of 30 MPa [1]. The AY sample has a value above that of monolithic AlN, while the AN and AZ samples have values below that of monolithic AlN. The fracture SEM micrograph of AY (Figure 2d) shows delimited grains of AlN, indicating that the Y_2_O_3_ causes intergranular fracture and thus improves the toughness. This increment in toughness can be observed in the interaction of cracks with elastic interfaces.

### 2.2. TiB_2_ Composites

#### 2.2.1. Crystalline Phases

**Figure 3:**
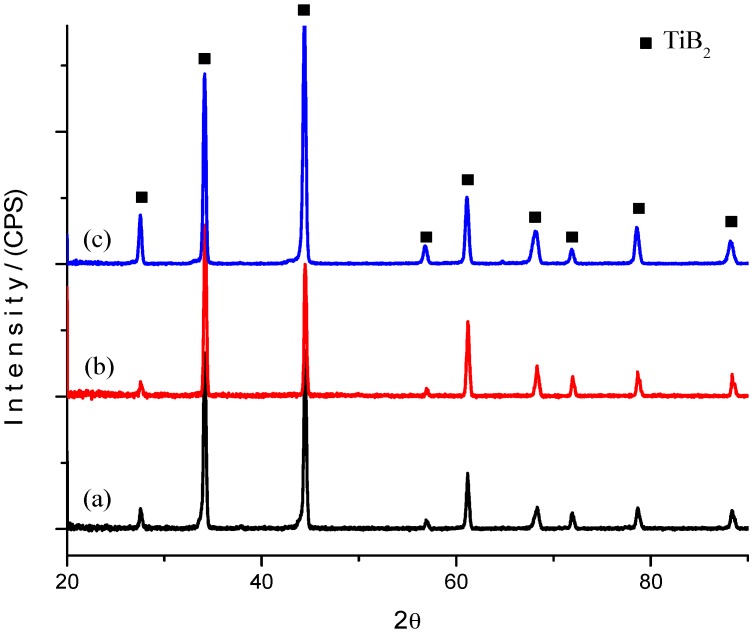
XRD patterns of sintered TiB_2_ samples with different oxides: (**a**) TN; (**b**) TY; (**c**) TZ.

Figure 3 shows the XRD patterns of the TiB_2_-based composites after sintering at 1950 °C. In this case, the XRD patterns were obtained from the polished surface of the specimen. As shown, the phase composition of TiB_2_-Nb_2_O_5_ (TN), TiB_2_-Y_2_O_3_ (TY) and TiB_2_-ZrO_2_ (TZ) indicated that the main phase for the as-sintered composite is TiB_2_.

#### 2.2.2. Microstructural Analysis

**Figure 4:**
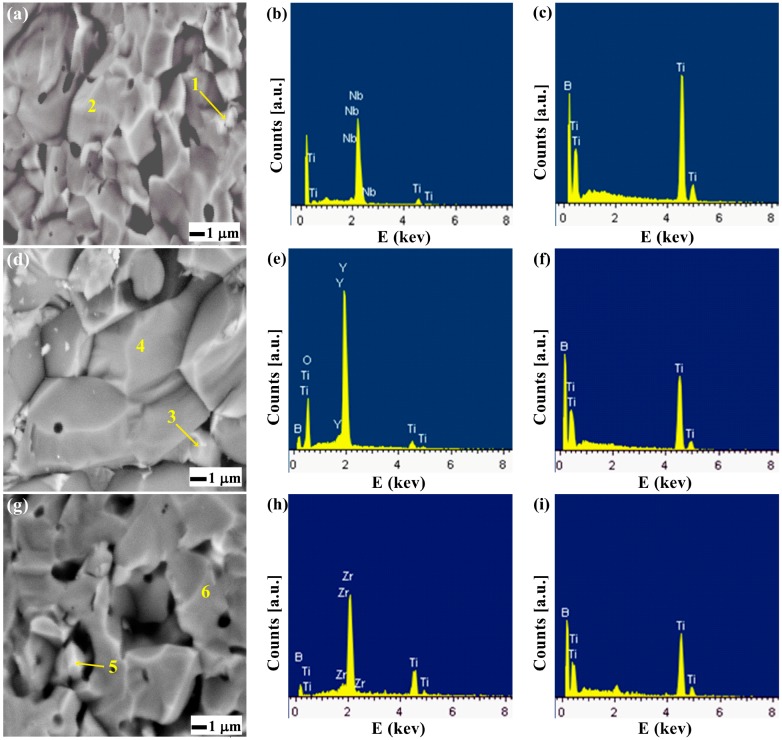
Secondary electron SEM images of fracture surfaces of TiB_2_ specimens sintered: (**a**) TN; (**d**) TY; (**g**) TZ; EDS spectra of secondary phases: (**b**) analysis of area 1; (**e**) analysis of area 3; (**h**) analysis of area 5; (**c**,**f**,**i**) are from the matrix grains.

Figure 4 shows the SEM images of the fracture surfaces of TiB_2_ specimens at 1950 °C and the microanalyses of EDS of the secondary phase in TN, TY and TZ composites. The SPS-sintered TiB_2_ specimens primarily showed an intra-granular fracture mode. The microstructures are composed of TiB_2_ grains and secondary phases. Nb_2_O_5_ was found in TN, and ZrO_2_ was found in TZ. The EDS characterization showed that the secondary phase consists of B, Ti, O and Y. According to the literature, in the TY sample, the formation of Y_3_BO_6_ is detected at the interface of the Y_2_O_3_ and TiB_2_ phases [20]. It is thought that this intergranular reaction promotes particle rearrangement for densification and/or the disappearance of pores under the applied pressure during sintering.

#### 2.2.3. Mechanical Properties

The mechanical properties (Table 3) of the composites were compared with the hardness of monolithic TiB_2_ (25 GPa) [1]. The hardness increased approximately 10% in the TN sample, 9% in the TY sample and 6% in the TZ sample (Table 3). This increased due to the addition of oxides. The Vickers hardness measurement in the TZ sample showed that the hardness was lower compared with the TN and TY samples, due to the volume expansion in the lattice of the material, reducing the relative density (Table 1) and Vickers hardness (Table 3), as the sintering temperature does influence the relative density and hardness. According to the literature, the relative density and hardness tend to increase as the sintering temperature increases, i.e., from 1500 °C to 1800 °C. When the sintering temperature exceeded 1700 °C, the relative density increased at a rapid rate and the hardness increased slowly.

The measured toughness values, obtained with an indentation a load of 20 kg, are reported in Table 3. Zhang et al. [21] reported a fracture toughness of 5.2 MPa·m^1/2^ for monolithic TiB_2_ ceramics sintered at 1800 °C with a holding pressure of 50 MPa. The rise of the sintering temperature increased the density and dropped the porosity of the TiB_2_ composites. Smaller grains lead to more grain boundaries, which can impede the propagation of cracks via crack deflection, thus absorbing energy for microcrack expansion. Therefore, the fracture toughness decreases as the grain size increases. Comparing the values obtained in the present work with the fracture toughness of 5.2 MPa·m^1/2^ for monolithic TiB_2_, the fracture toughness increased approximately 5% in the TN sample, 3% in the TY sample and 7% in the TZ sample. Improving fracture toughness is interesting if the values obtained here agree with others reported in the literature for similar materials. For the TZ composite, the densification temperature plays an important role in the fracture toughness due to the cooling process. There may be a phase transformation of *t*-ZrO_2_ to *m*-ZrO_2_ [16].

## 3. Materials and Methods

### 3.1. Raw Materials

Commercially available AlN powder (grade A100 WR, Advanced Refractory Technologies, Buffalo, NY, USA) and TiB_2_ powder (98.64%, Storchem, Inc., Burlington, ON, Canada) were used as raw materials. Nb_2_O_5_ powder (99.5%, Strem Chemicals, Newburyport, MA, USA), ZrO_2_ powder (99%, Strem Chemicals) and Y_2_O_3_ powder (99.99%, Strem Chemicals) were used as sintering additives. The sample compositions are shown in Table 4.

### 3.2. Experimental Procedure

Powders were ball milled for 4 h in acetone to disperse and homogenize the mixtures. After mixing and drying, the mixture was poured into a graphite die 20 mm in diameter and then sintered at 1800 °C under a nitrogen atmosphere for AlN samples and 1950 °C under a argon atmosphere for TiB_2_ samples in an SPS equipment (Dr. SINTER SPS-1050-CE). During the SPS process, both heating and cooling rate were controlled at 150 °C/min for all samples. A pressure of 40 MPa from the beginning to the end of the sintering cycle was applied to the samples with AlN and 50 MPa for the samples with TiB_2_. After 15 min holding time, samples 20 mm in diameter and 5.5–6.3 mm in thickness were obtained. The sintering temperature was selected after previous sintering cycles that evaluated the density and shrinkage behavior using the SPS internal dilatometer.

### 3.3. Characterization and Methods

Once the SPS process was completed, the densities of samples were measured in water according to Archimedes’ principle. The relative density was calculated based on the densities of AlN (3.26 g/cm^3^), TiB_2_ (4.52 g/cm^3^), Nb_2_O_5_ (4.47 g/cm^3^), Y_2_O_3_ (5.01 g/cm^3^), ZrO_2_ (6.05 g/cm^3^), Nb_4_N_3_ (8.43 g/cm^3^), YAlO_3_ (5.35 g/cm^3^) and ZrO (7.22 g/cm^3^) according to the rule of mixtures. The hardness (H_V_) of the samples was measured at room temperature by the Vickers diamond indentation method; 10 indentations were made on each sample under a load of 4.9 N and a dwell time of 15 s. The indentation fracture toughness (K_IC_) of the samples was based on the length of the cracks originating from the edges of the indentation marks using the equations given by Evans and Charles [22] after being carefully polished by standard diamond polishing techniques down to 1 μm finish. For statistical purposes, 10 indentations were carried out for each sample under a load of 196 N and a dwell time of 15 s.

The crystalline phases were characterized by X-ray diffraction (XRD, Bruker 08 Advance) with Cu Kα radiation. The fracture surfaces of the samples were observed by a scanning electron microscope (JEOL JSM-7600F, Akishima, Tokyo, Japan) equipped with energy-dispersive spectroscopy (EDS) with a ultra-thin window (UTW) detector to examine the microstructure.

## 4. Conclusions

Dense AlN and TiB_2_ composites with Nb_2_O_5_, Y_2_O_3_ and ZrO_2_ were successfully achieved by SPS. Sintering at 1800 °C under a pressure of 40 MPa in nitrogen for AlN composites was successfully achieved. A temperature of 1950 °C under a 50 MPa pressure in argon was used for sintering TiB_2_ composites. XRD analysis of AlN composites in the sintering process showed that Nb_4_N_3_ was formed in the AN sample, YAP was produced in the AY sample and ZrO in the AZ sample. XRD analysis of the TiB_2_ composites showed only TiB_2_. The Vickers hardness increased with the amount of oxide additives, exhibiting a hardness between 11 and 14 GPa for the AlN composites and between 26 and 28 GPa for TiB_2_ composites. The values of the hardness and toughness are higher than those reported in the literature.

## Figures and Tables

**Table 1:** Densities values of the produced composites.

Composites	Theoretical Density (g/cm^3^)	Measured Density (g/cm^3^)	Relative Density (%)
AlN-Nb_2_O_5_ (AN)	3.51	3.29	93.7
AlN-Y_2_O_3_ (AY)	3.36	3.28	97.6
AlN-ZrO_2_ (AZ)	3.45	3.33	96.5
TiB_2_-Nb_2_O_5_ (TN)	4.52	4.35	96.2
TiB_2_-Y_2_O_3_ (TY)	4.54	4.39	96.7
TiB_2_-ZrO_2_ (TZ)	4.59	4.23	92.2

**Table 2:** Results of the mechanical properties for the AlN composites.

Samples	Hv (GPa)	ΔHv (%) ^a^	K_IC_ (MPa·m^1/2^)	ΔK_IC_ (%) ^a^
AlN	10.6 ^b^	-	3.2 ^b^	-
AN	14.19 ± 1.43	34	2.82 ± 0.14	−12
AY	11.45 ± 0.39	8	3.40 ± 0.47	6
AZ	14.06 ± 1.26	33	2.86 ± 0.11	−11

^a^ ΔHv and ΔK_IC_ indicate the variation of the composites hardness and fracture toughness with respect to the AlN values; ^b^ Values taken from the reference [1].

**Table 3:** Results of the mechanical properties for the TiB_2_ composites.

Samples	Hv (GPa)	ΔHv (%) ^a^	K_IC_ (MPa·m^1/2^)	ΔK_IC_ (%) ^a^
TiB_2_	25 ^b^	-	5.2 ^c^	-
TN	27.52 ± 1.75	10	5.45 ± 0.15	5
TY	27.28 ± 3.54	9	5.33 ± 0.22	3
TZ	26.50 ± 2.45	6	5.54 ± 0.31	7

^a^ ΔHv and ΔK_IC_ indicate the variation of the composites’ hardness and fracture toughness with respect to the TiB_2_ values. ^b,c^ Values taken from References [1,21], respectively.

**Table 4:** Compound compositions and sintering parameters.

Samples	Starting Powders (wt %)					Processing Conditions		
	AlN	TiB_2_	Nb_2_O_5_	Y_2_O_3_	ZrO_2_	Sintering Temperature (°C)	Holding Time (min)	Pressure (MPa)
AN	95.24	-	4.76	-	-	1800	15	40
AY	95.24	-	-	4.76	-	1800	15	40
AZ	95.24	-	-	-	4.76	1800	15	40
TN	-	95.24	4.76	-	-	1950	15	50
TY	-	95.24	-	4.76	-	1950	15	50
TZ	-	95.24	-	-	4.76	1950	15	50
